# The immune-related role of BRAF in melanoma

**DOI:** 10.1186/1479-5876-13-S1-K19

**Published:** 2015-01-15

**Authors:** Sara Tomei, Davide Bedognetti, Valeria De Giorgi, Michele Sommariva, Sara Civini, Jennifer Reinboth, Muna Al Hashmi, Maria Libera Ascierto, Qiuzhen Liu, Ben D Ayotte, Andrea Worschech, Lorenzo Uccellini, Paolo A Ascierto, David Stroncek, Giuseppe Palmieri, Lotfi Chouchane, Ena Wang, Francesco M Marincola

**Affiliations:** 1 Infectious Disease and Immunogenetics Section (IDIS), Department of Transfusion Medicine, Clinical Center and Trans-NIH Center for Human Immunology (CHI), National Institutes of Health, Bethesda, Maryland, USA; 2 Department of Genetic Medicine, Weill Cornell Medical College in Qatar, PO Box 24144, Doha, Qatar; 3 Sidra Medical and Research Center, P.O. Box 26999, Doha, Qatar; 4 Department of Biomedical Sciences for Health, Universita degli Studi di Milano, Milan, Italy; 5 Cell Processing Section, Department of Transfusion Medicine, Clinical Center and Trans-NIH Center for Human Immunology (CHI), National Institutes of Health, Bethesda, Maryland, USA; 6 Department of Biochemistry, Biocenter, University of Wuerzburg, Wuerzburg 97074, Germany; 7 Genelux Corporation, San Diego Science Center, San Diego, California, USA; 8 Center of Excellence for Biomedical Research (CEBR), University of Genoa, Italy; 9 Department of Biology, Northern Michigan University, Marquette, MI, USA; 10 Institute of Infectious and Tropical Diseases, University of Milan, L. Sacco Hospital, Milan, Italy; 11 Istituto Nazionale Tumori Fondazione “G. Pascale”, Via G. Semmola, Naples, Italy; 12 Institute of Biomolecular Chemistry, National Research Council, Sassari, Italy

## Background

In the recent years there have been major advances in the field of cancer immunology and the existence of a dichotomy between immunologically active and quiescent tumor phenotypes has been recognized in several cancers. The activation of a Th1 immune signature has been shown to confer better prognosis and likelihood to respond to immunotherapy. However, whether such dichotomy depends on the genetic make-up of individual cancers is not known yet. In melanoma, BRAF and NRAS mutations are commonly acquired during tumor progression. Although the oncogenic potential of BRAF and NRAS alterations has been attributed to reduced apoptosis, increased invasiveness and increased metastatic behavior, the role of BRAF and NRAS in the immunological landscape of cutaneous melanoma has been poorly investigated and the effects of BRAF and NRAS mutations on global gene expression remain to be understood. We explored the role of BRAF and NRAS mutations at the transcriptome level and in influencing the immune phenotype (based on a classification previously identified by our group).

## Materials and methods

One-hundred-thirteen pre-treatment snap frozen tumor biopsies were collected from patients treated at the Surgery Branch, NCI (Bethesda, Maryland) and processed for DNA and RNA isolation. Each sample underwent microarray analysis and BRAF and NRAS genotyping. Allele-specific PCR was also performed in order to exclude low-frequency mutations. Fifteen melanoma cell lines were also tested for BRAF and NRAS mutation by Sanger sequencing and RNA-sequencing.

## Results

Comparison between BRAF and NRAS mutant versus wild type samples identified mostly constituents or regulators of MAPK and related pathways. Initially, we postulated that there might be a common MAPK activation signature resulting from either BRAF or NRAS mutation; however, we found no overabundance of discriminatory genes for the combined group of samples displaying either BRAF or NRAS mutations. This suggests that the transcriptional consequences resulting from mutations of BRAF or NRAS might be different, although there was overlapping of some genes, presumably due to their differential capacity to receive input signals and transduce them through different effectors.

When testing gene lists discriminative of BRAF, NRAS and MAPK alterations, we found that 112 BRAF-specific transcripts were able to distinguish the two immune-related phenotypes already described in melanoma, with the poor phenotype associated mostly with BRAF mutation. Noteworthy, such association was stronger in samples displaying low BRAF mRNA expression. However, when testing NRAS mutation, we were not able to find the same association. Class comparison between BRAF mutant samples with high and low expression of the same gene identified 6296 transcripts. Functional interpretation analysis showed that these 6296 transcripts were associated to IL-2 and JAK/Stat signaling pathways, supporting the immunoregulatory role of BRAF. Additionally, fifteen melanoma cell lines were also tested by BRAF and NRAS DNA genotyping and RNA-sequencing. Interestingly, we found that among 8 cell lines BRAF mutated (V600E), three of them expressed BRAF at low level and may have preferential wild type allele selection at the RNA level.

## Conclusion

In conclusion we provide novel insights into the effect of BRAF and NRAS mutations on gene expression according to the immune classification. However, further deeper analyses are warranted to understand the mechanisms behind the association of BRAF mutation with a poor immune phenotype and also behind BRAF low expression and wild type allele selection at the RNA level.

